# Supplementary material for: Pangenome graphs improve the analysis of structural variants in rare genetic diseases
Source: Nat Commun. 2024 Jan 22;15:657. doi: 10.1038/s41467-024-44980-2 (PMC10803329; doi:10.1038/s41467-024-44980-2)
Supplement: Supplementary file 1 — Supplementary Information [file 41467_2024_44980_MOESM1_ESM.pdf]

Supplementary Information

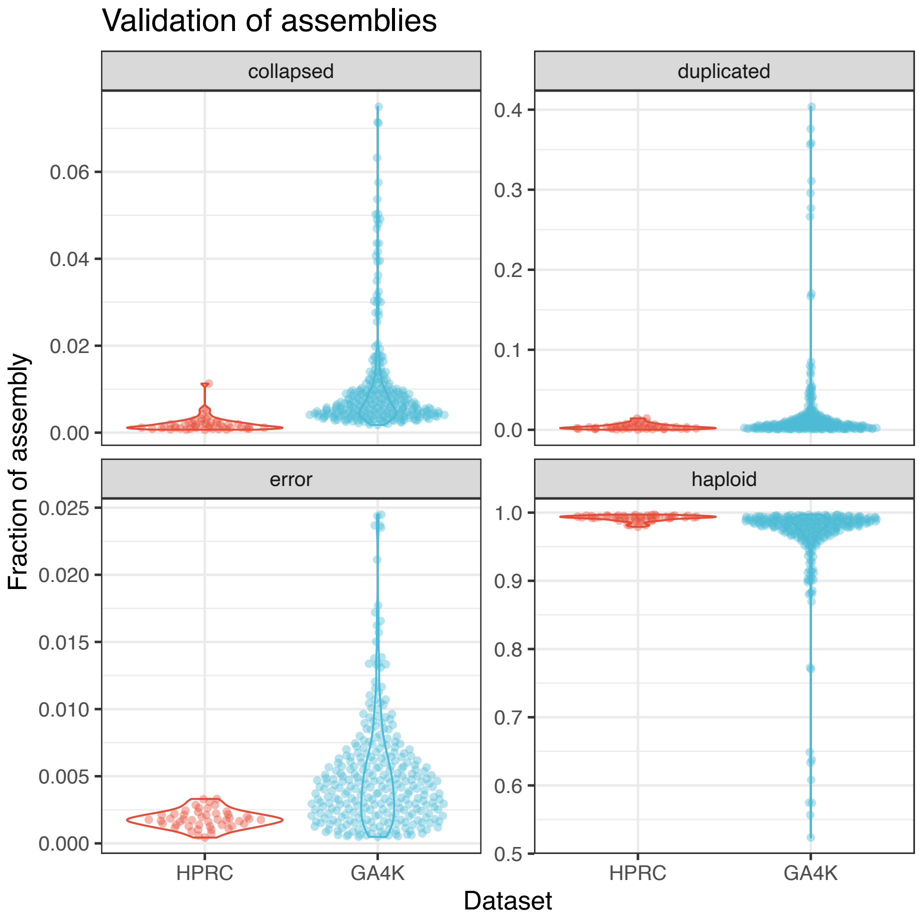

Supplementary Figure 1: Flagger results summarizing the proportion of each assembly that was labeled by Flagger as collapsed, duplicated, erroneously assembled, or properly assembled (haploid).

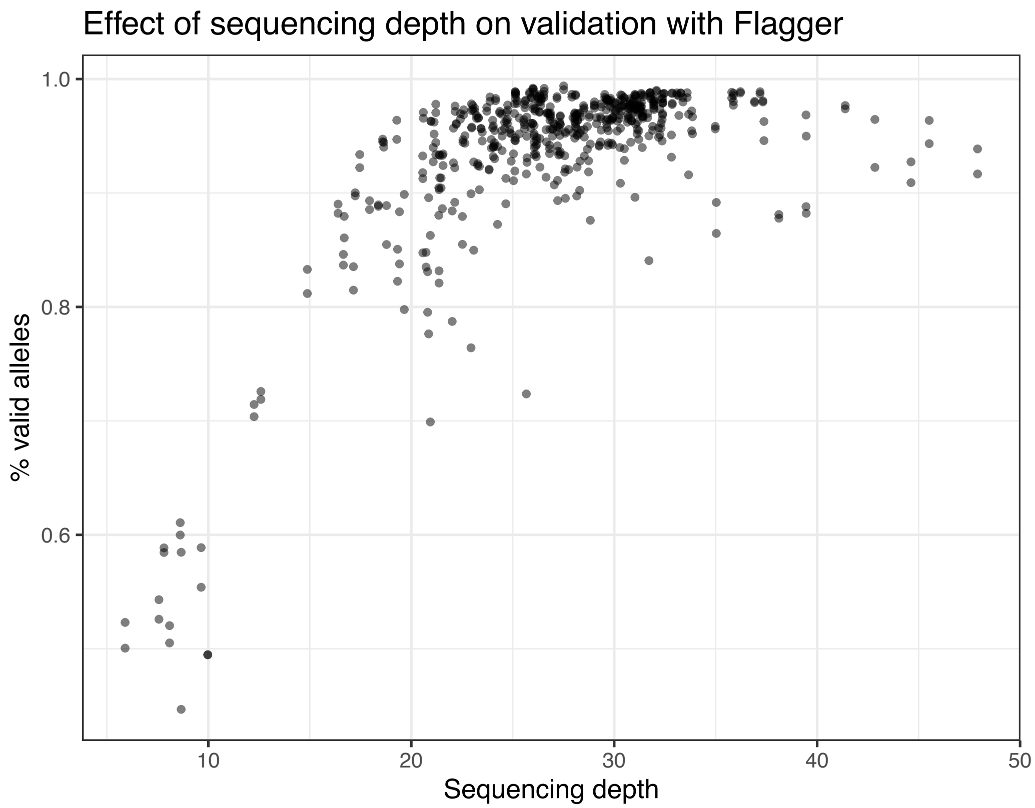

Supplementary Figure 2: The proportion of alleles that pass validation with Flagger in a genome versus it's sequencing depth.

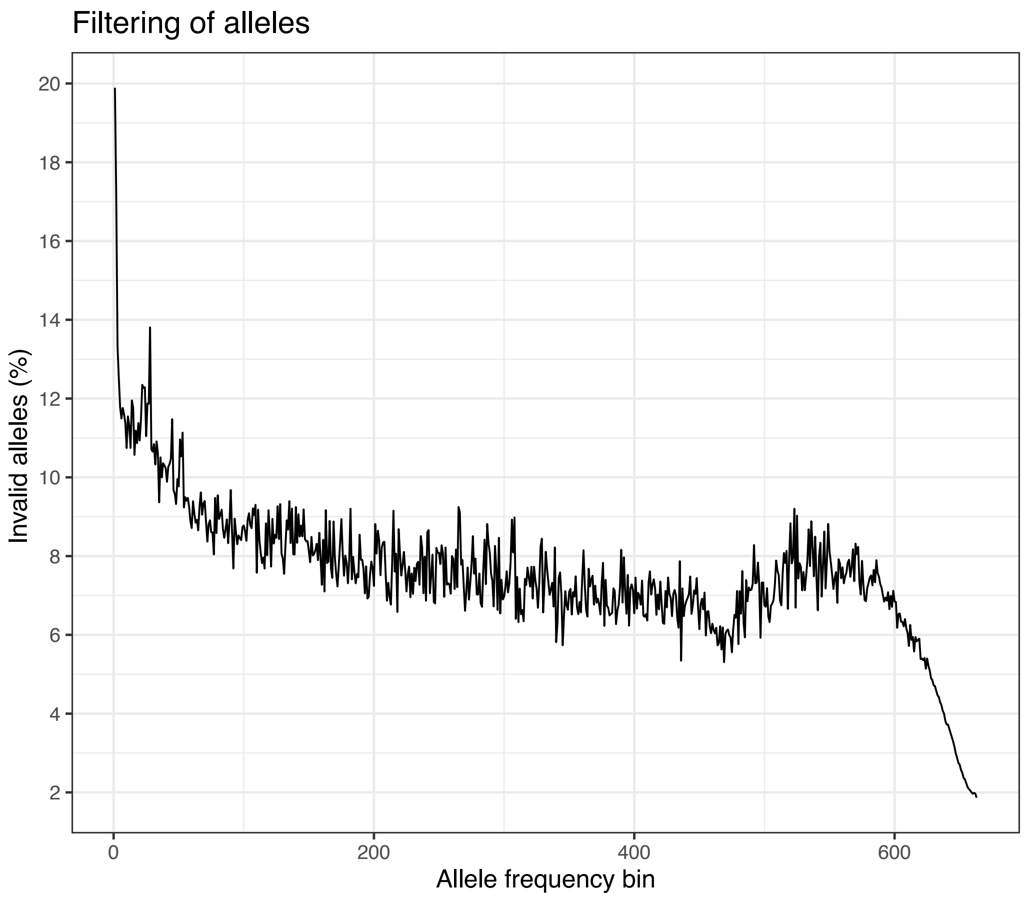

Supplementary Figure 3: The proportion of alleles that failed validation and were discarded, stratified by allele frequency.

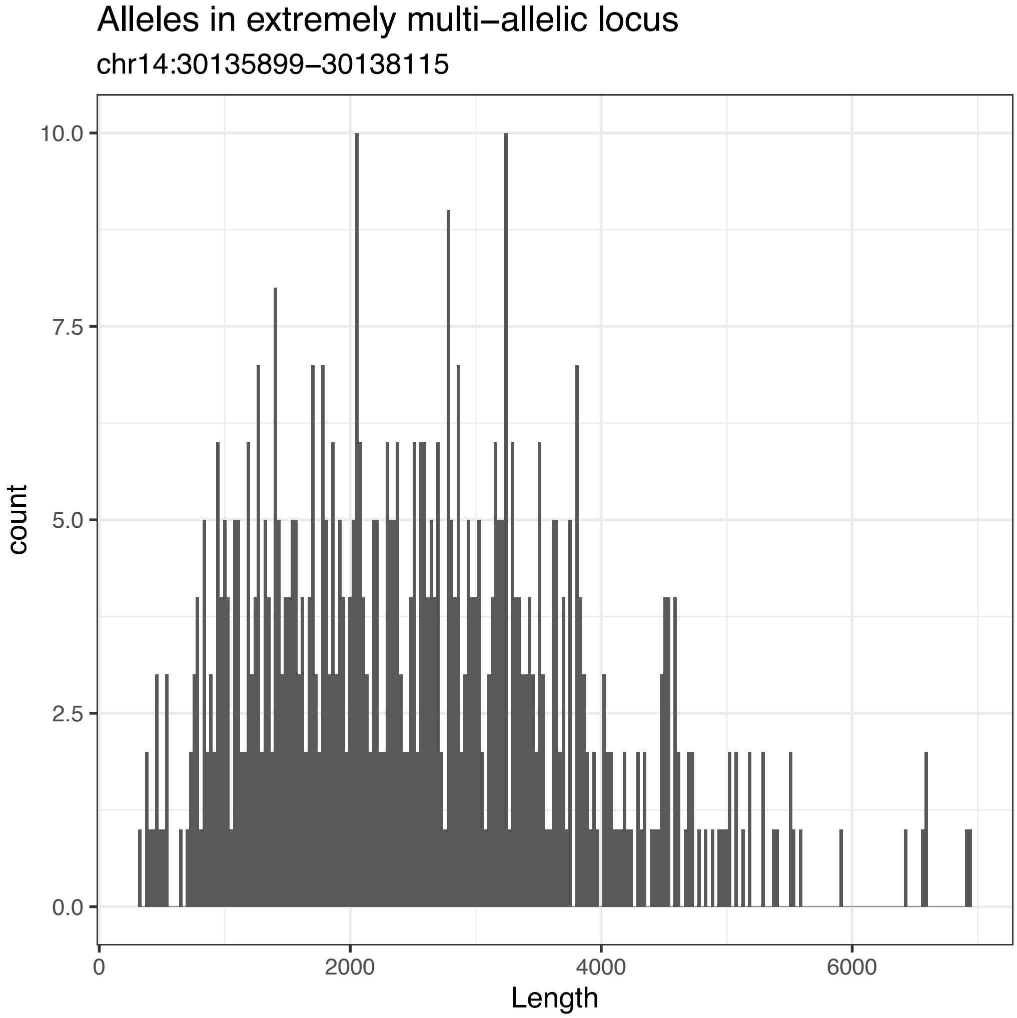

Supplementary Figure 4: Distribution of allele lengths in a highly polymorphic simple tandem repeat with a unit length of 27bp (CHM13v2 chr14:30135899-30138115) with 560 observed alleles.

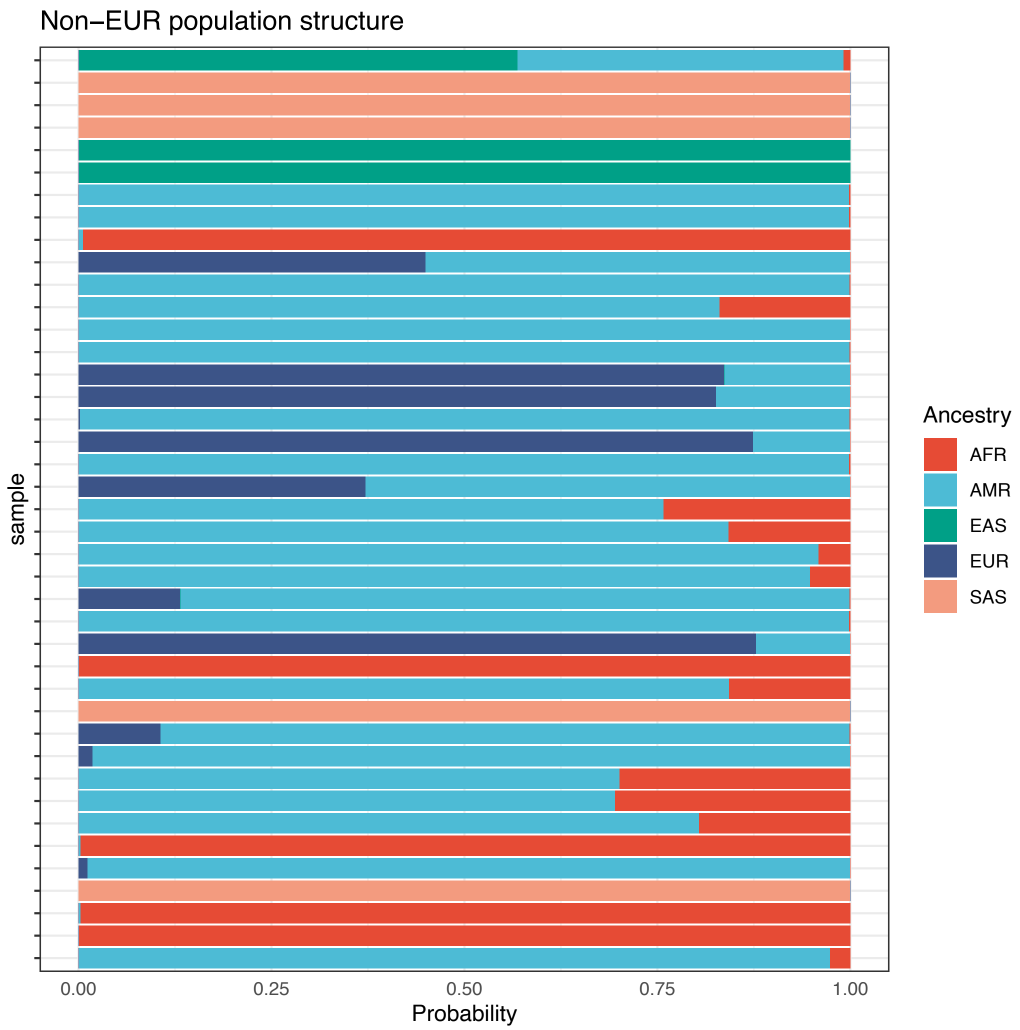

Supplementary Figure 5: Genome ancestry mapping results with somalier based on on SNVs, for genomes with more than 10% EUR contribution.

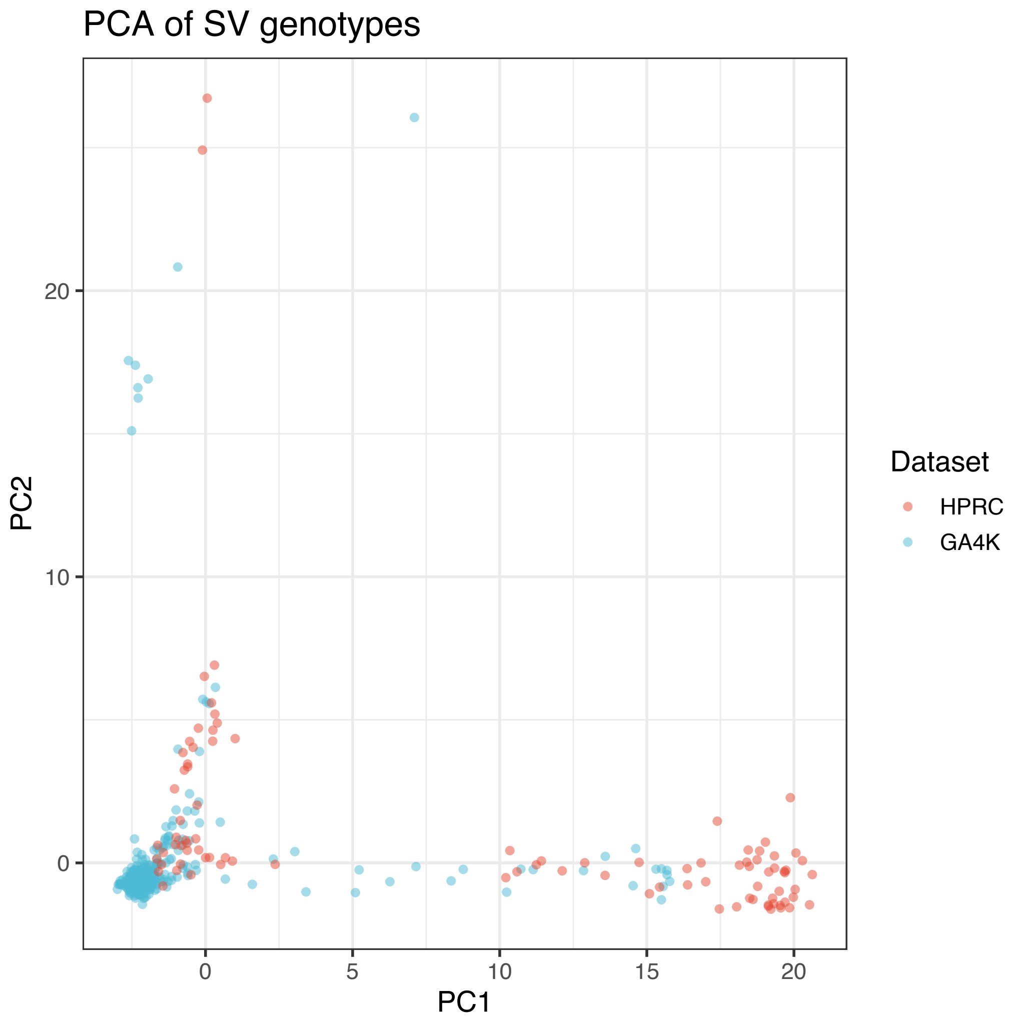

Supplementary Figure 6: Population structure obtained using a PCA projection of the full set of GA4K and HPRC SVs that were called with minigraph.

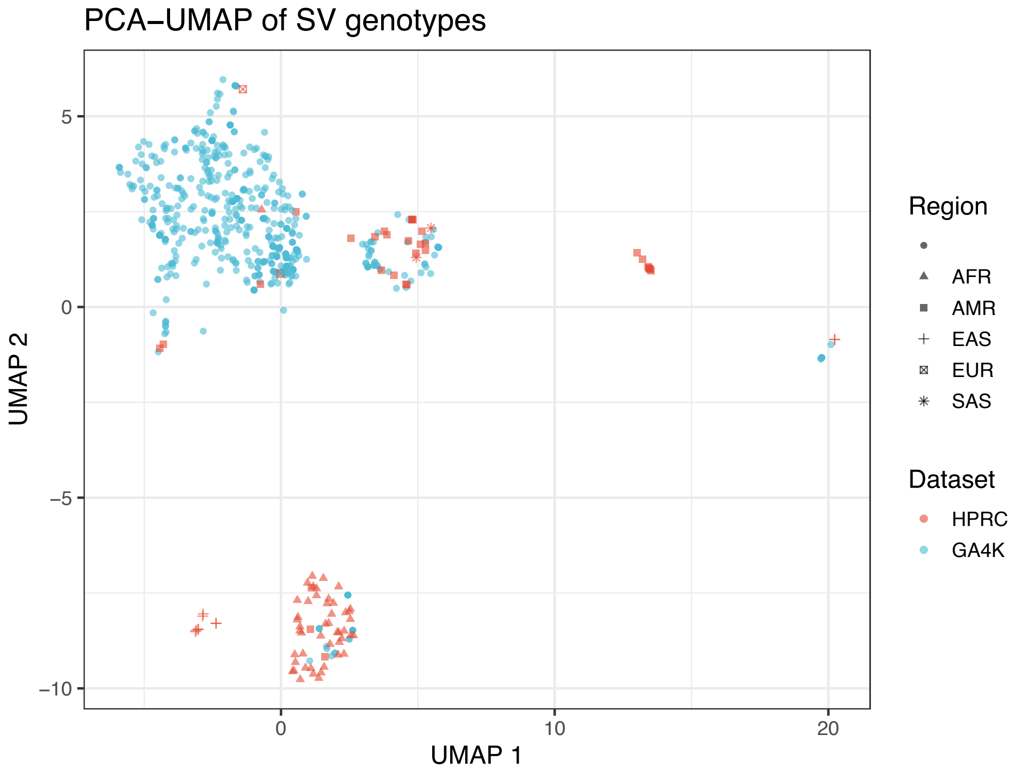

Supplementary Figure 7: Population structure obtained using a PCA-UMAP projection that was trained on a subset of GA4K and HPRC SVs that were called with minigraph.

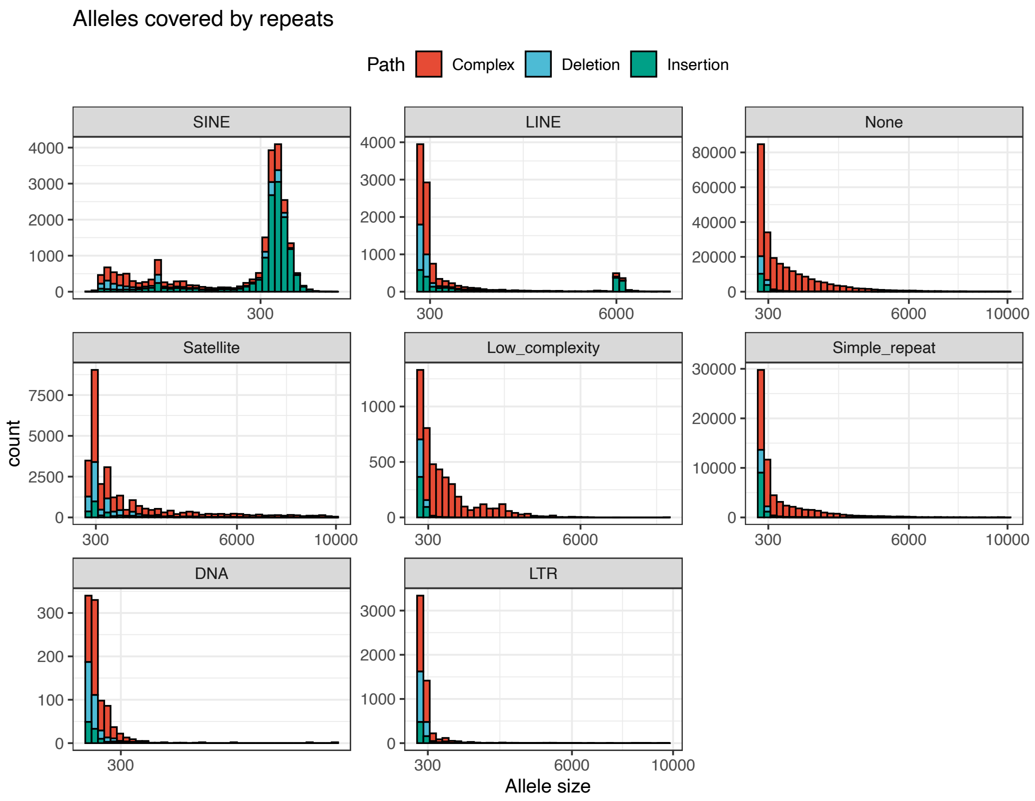

Supplementary Figure 8: Repeat spectrum in allele sequences in the pangenome, masked with RepeatMasker and stratified by allele type. Only alleles that are 80% covered by the annotation over their span are shown.

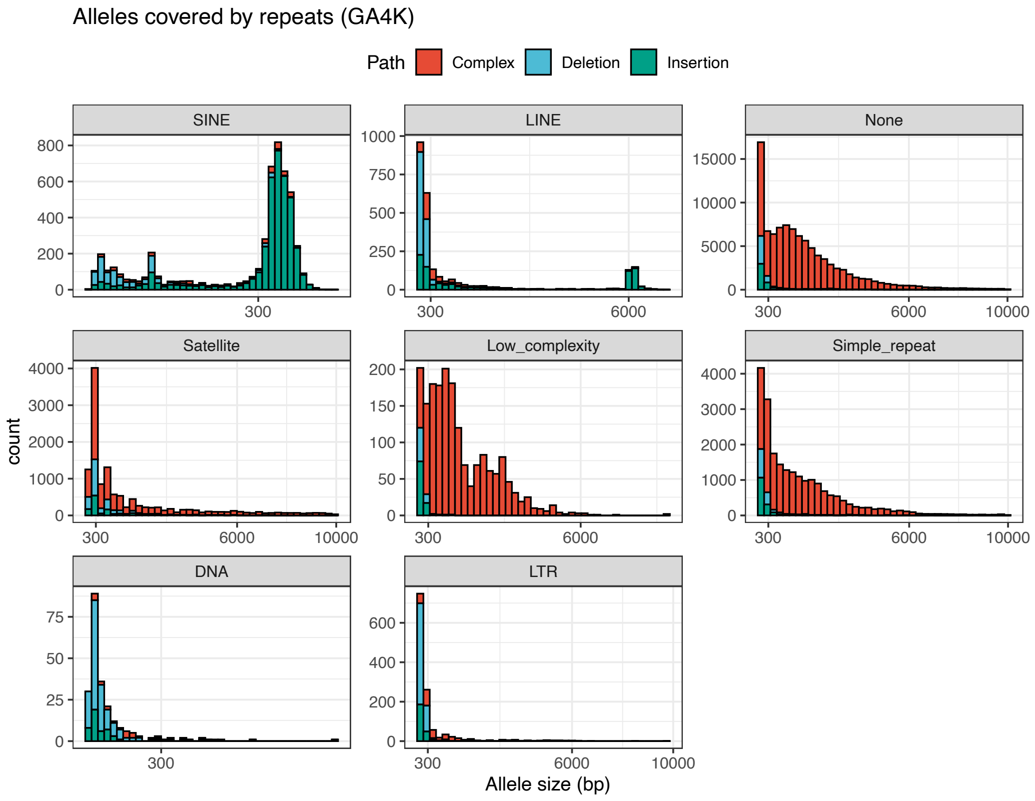

Supplementary Figure 9: Repeat spectrum in allele sequences that are unique to GA4K, masked with RepeatMasker and stratified by allele type. Only alleles that are 80% covered by the annotation over their span are shown.

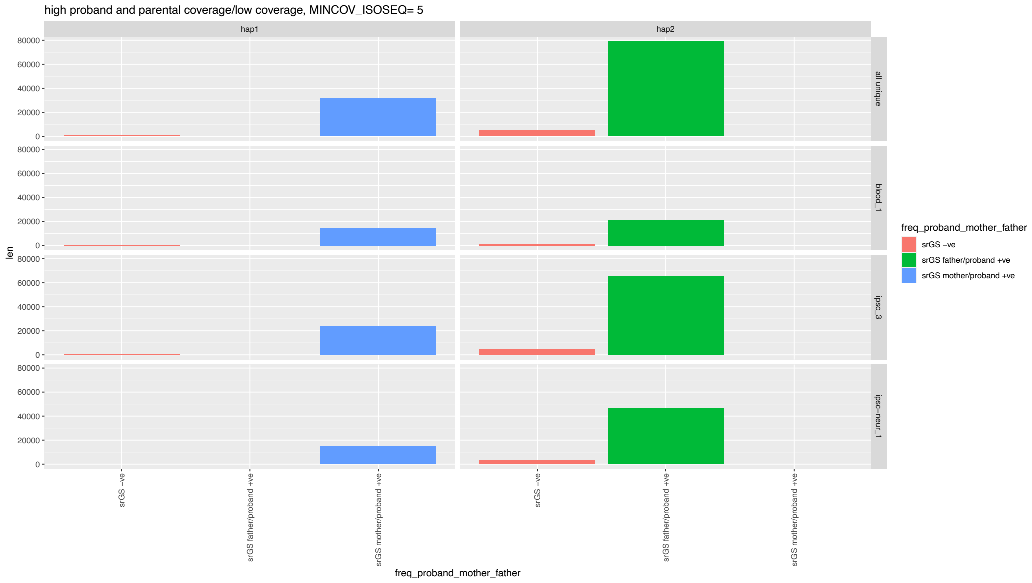

Supplementary Figure 10: Span of sequence that is covered by short-reads and Iso-Seq reads in contigs that are in a proband's assembly but that are not anchored in the genome graph.

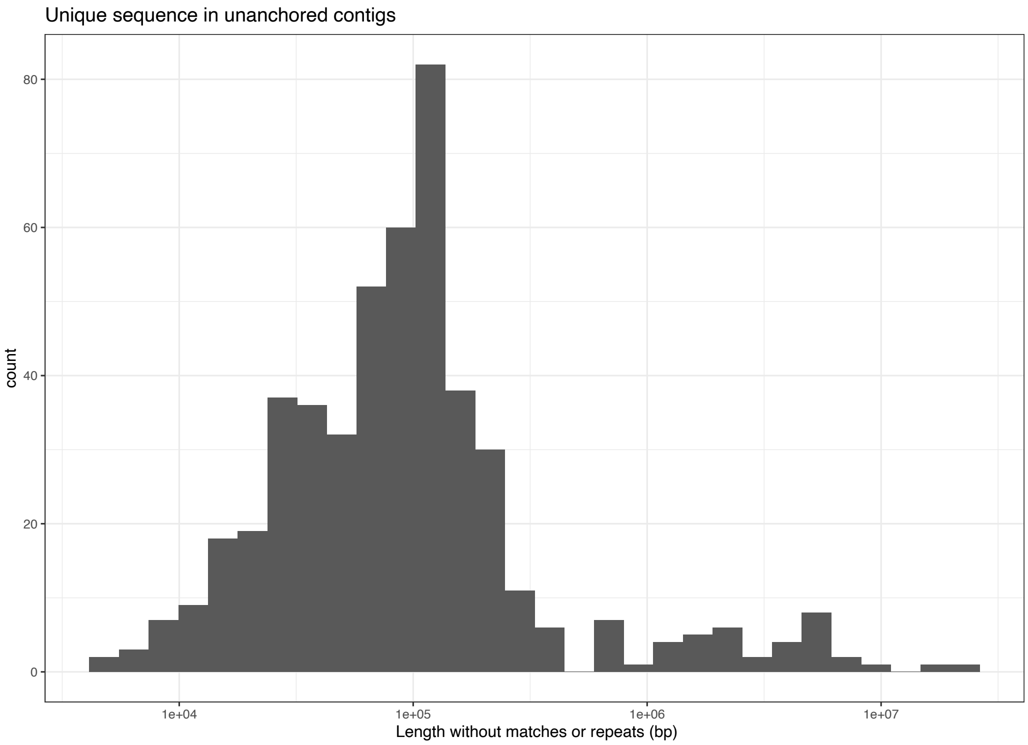

Supplementary Figure 11: Amount of sequence in unanchored contigs in each genome that does not map to chm13v2.0 and is not covered by repeats.

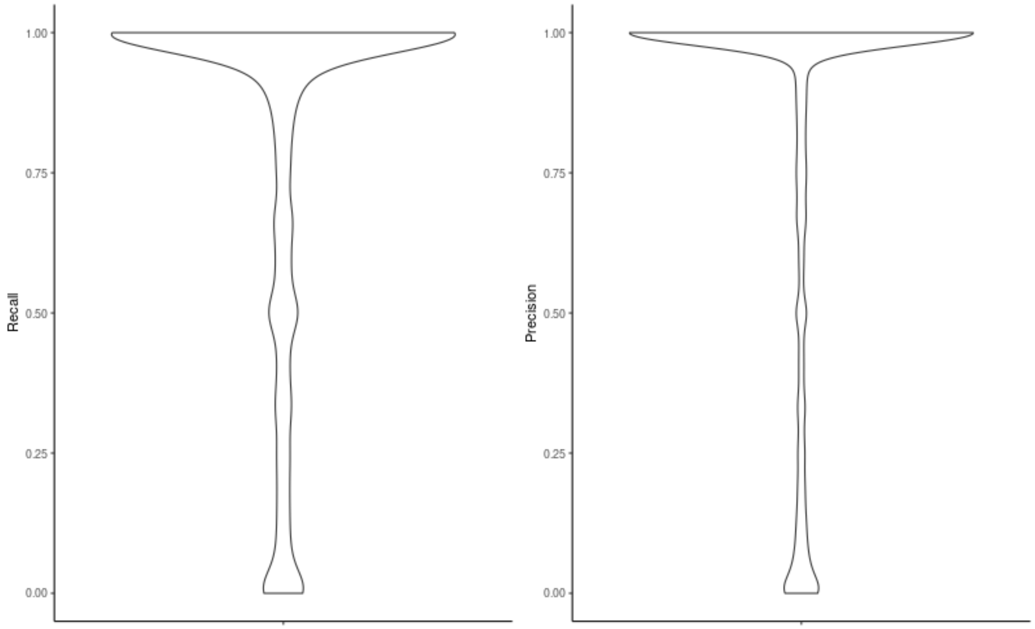

(A) (B)

Supplementary Figure 12: A) Recall and B) precision density distribution of minigraph genotypes against PBSV for figure 3A.

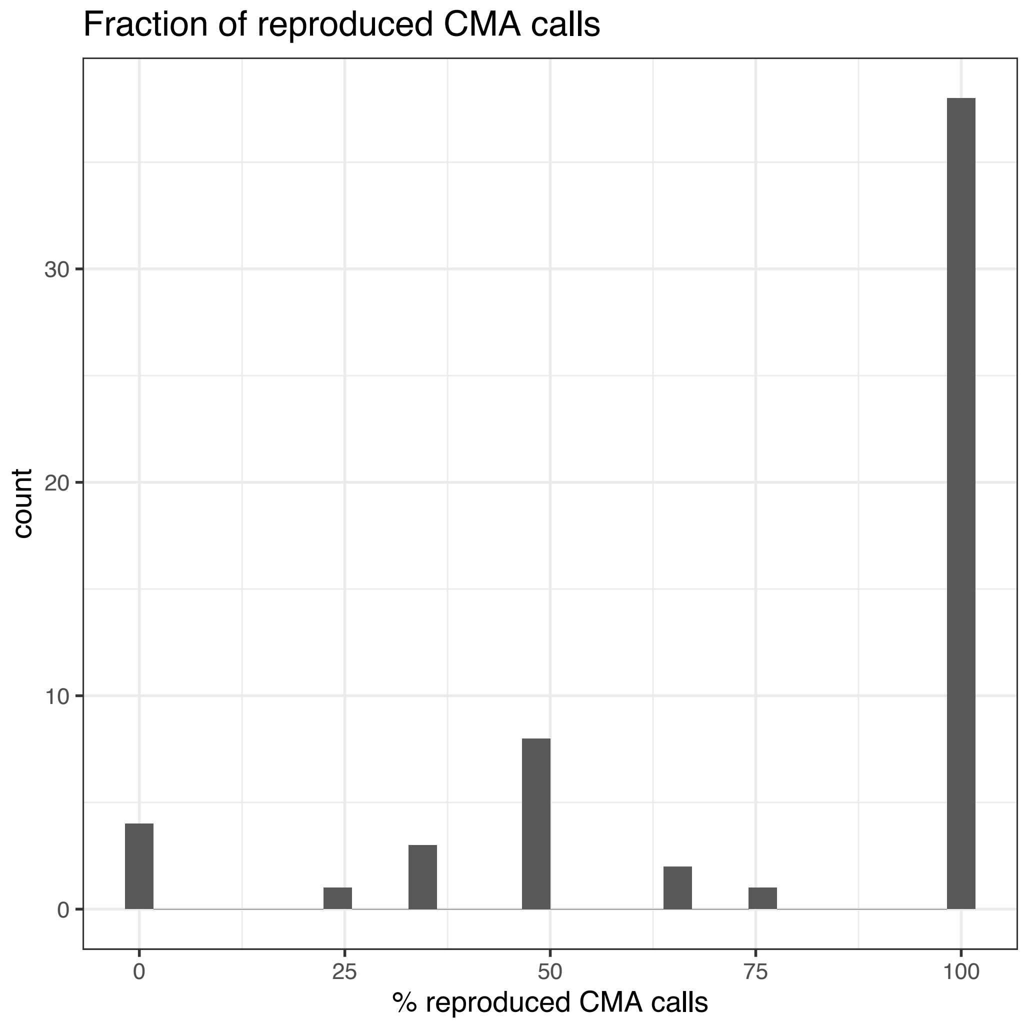

Supplementary Figure 13: Fraction of Chromosomal Microarray (CMA) SV calls reproduced by minigraph across CMA samples.

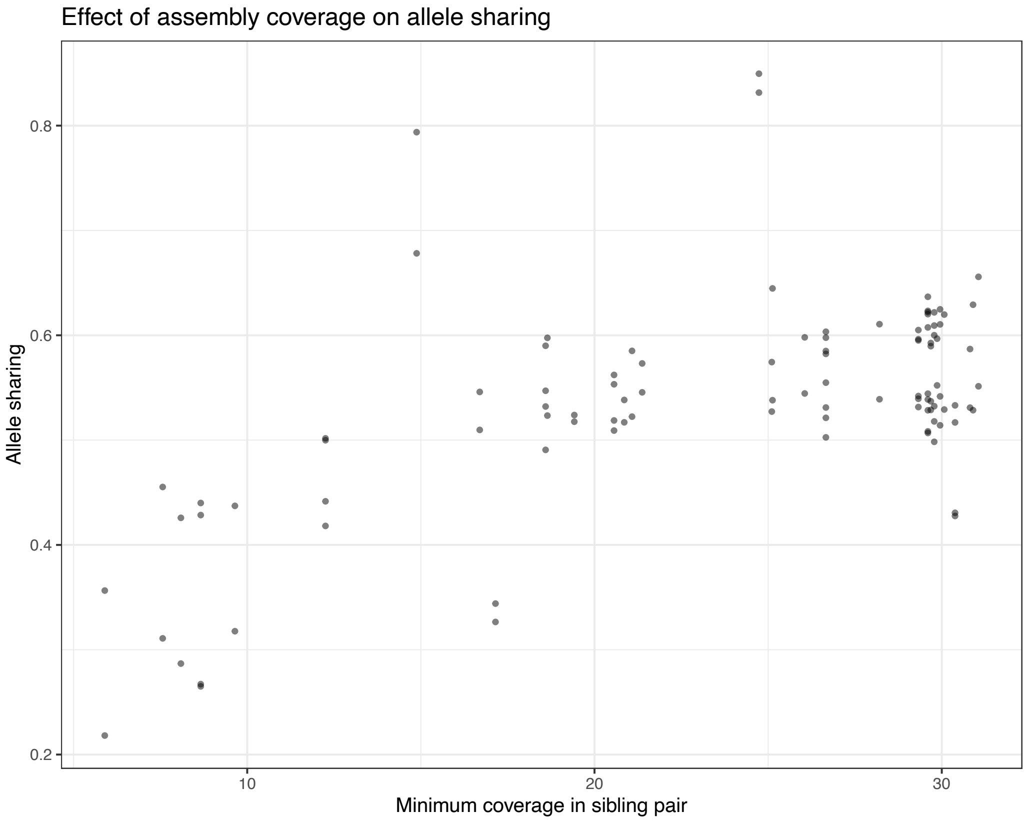

Supplementary Figure 14: The proportion of allele sharing between pairs of siblings versus the lowest sequencing depth of the genome pair.

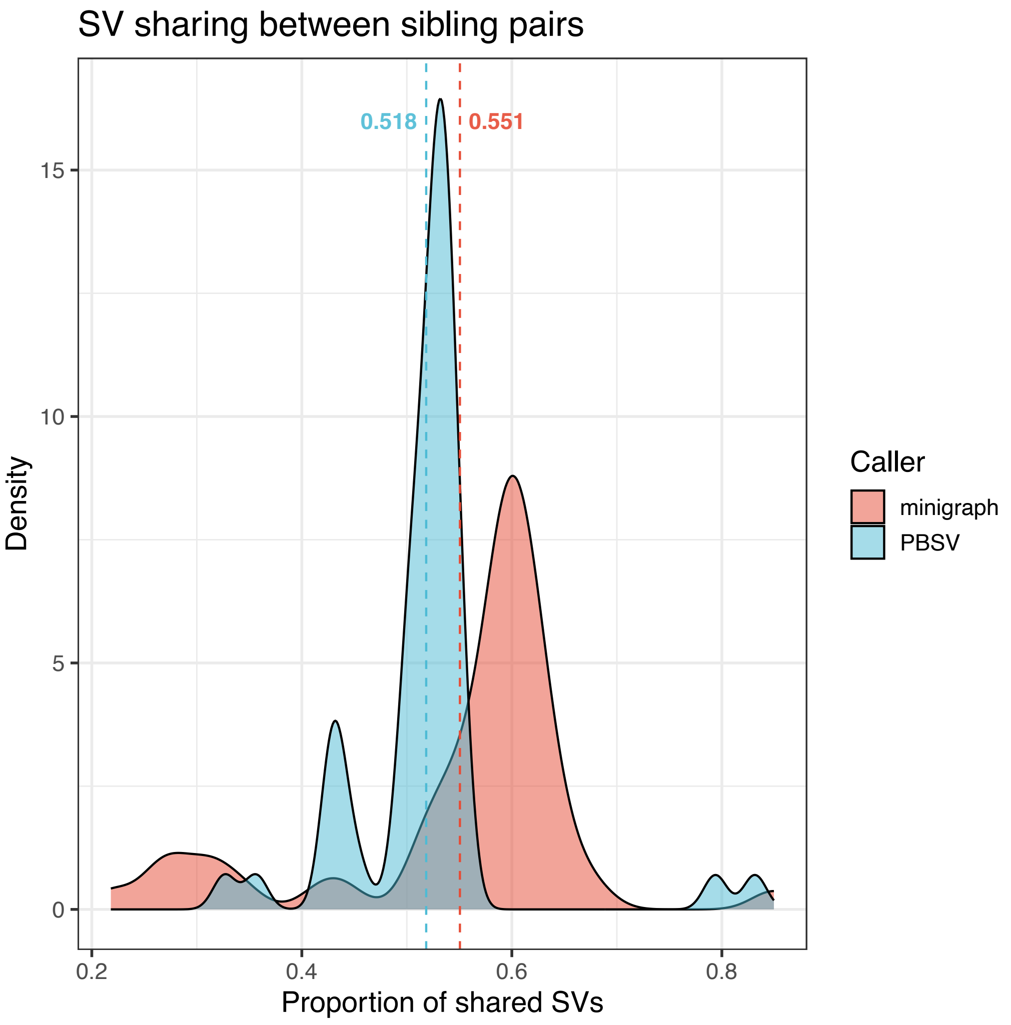

Supplementary Figure 15: Allele sharing distribution between siblings in GA4K (twins excluded and low coverage pairs included), calculated with PBSV versus minigraph.

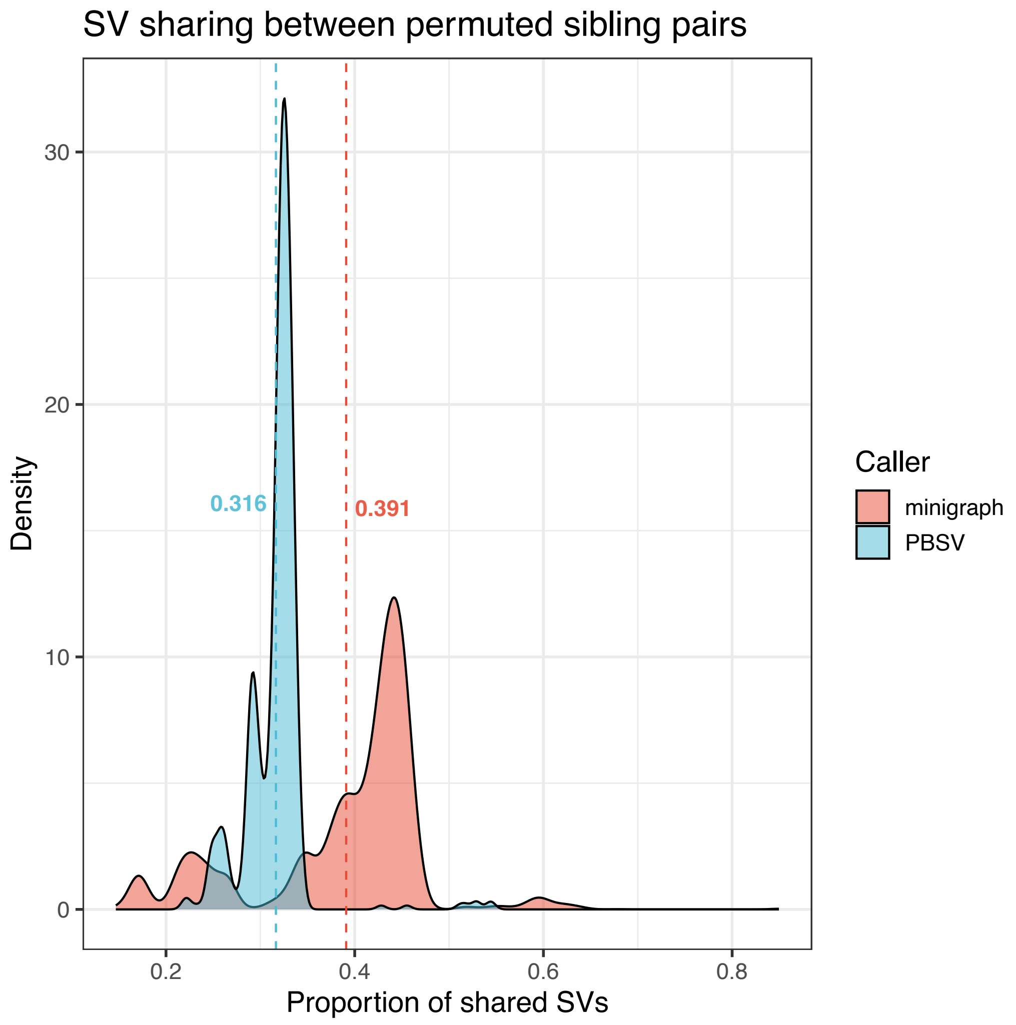

Supplementary Figure 16: Allele sharing distribution between randomly permuted sibling pairs in GA4K, calculated with PBSV and SURVIVOR versus minigraph.

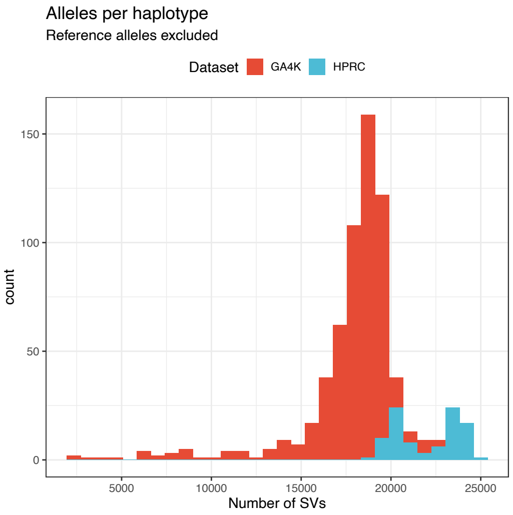

(A)

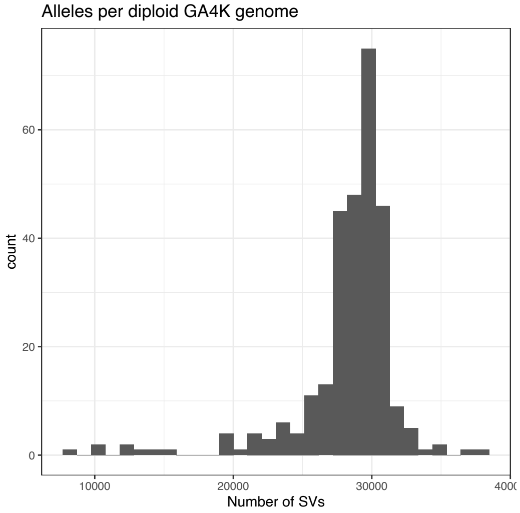

(B)

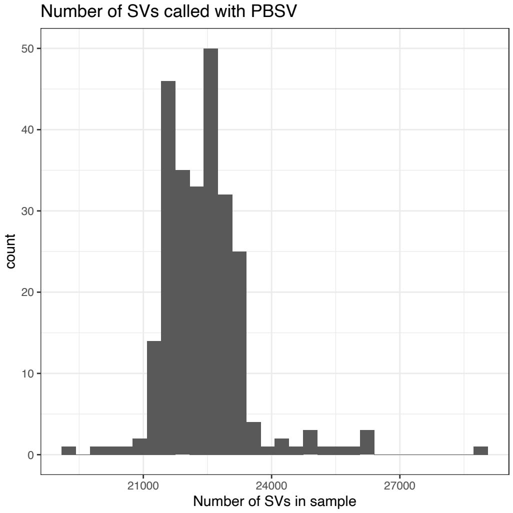

(C)

Supplementary Figure 17: A) Distribution of non-reference alleles per haplotype, stratified by HPRC and GA4K cohorts, B) per diploid genome in GA4K. C) Number of SVs called by PBSV per diploid genome.

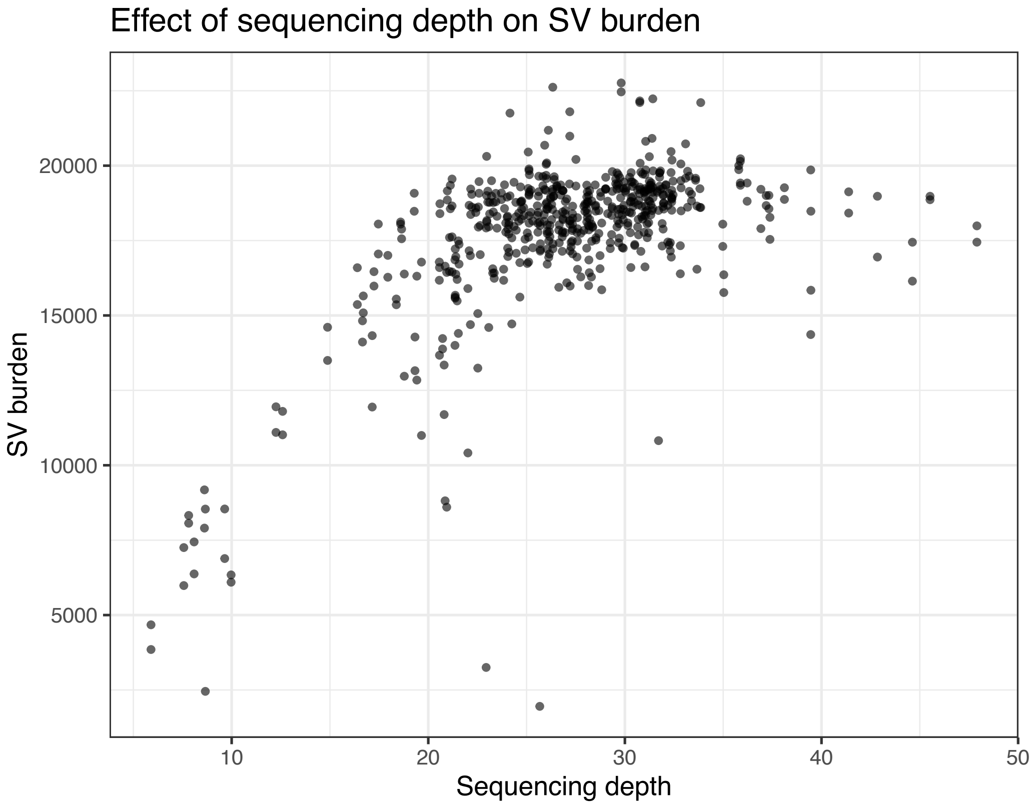

Supplementary Figure 18: SV burden of each genome in GA4K vs the sequencing depth of the genome.

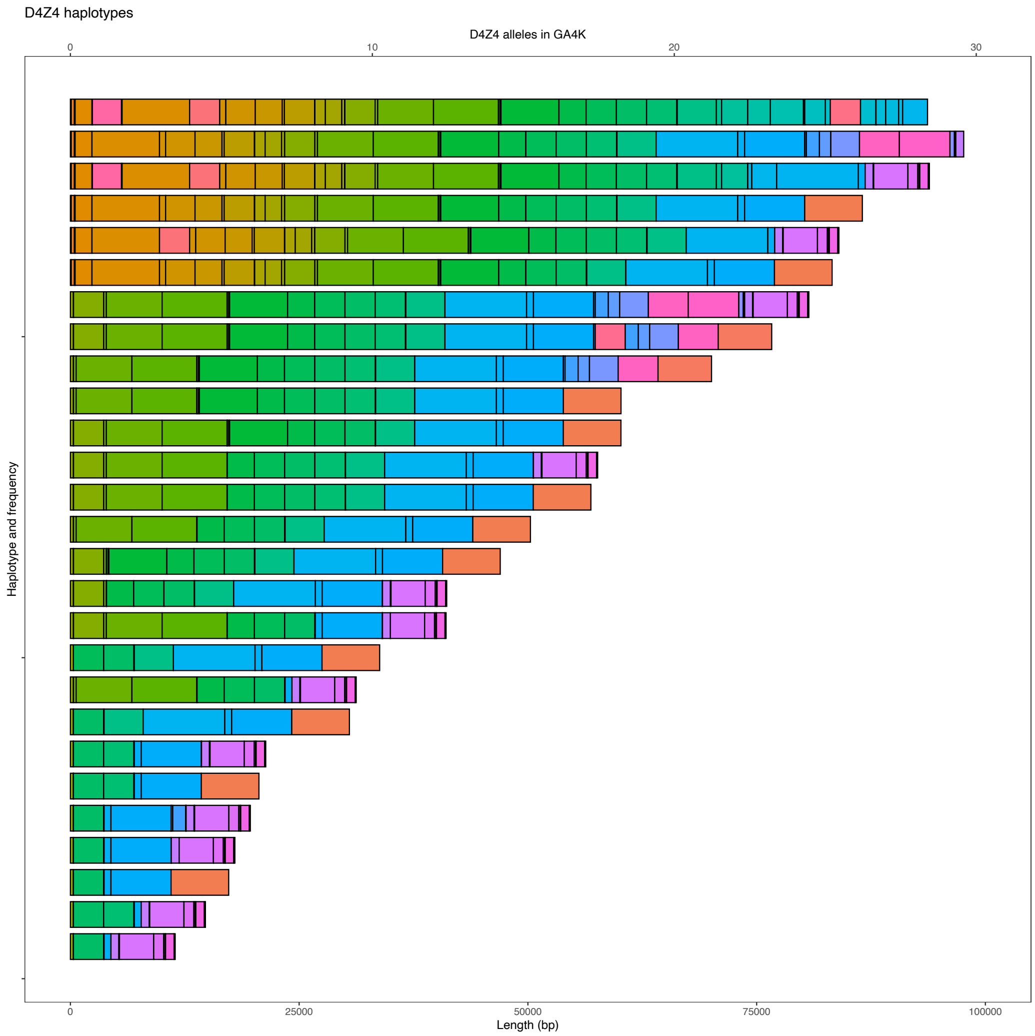

Supplementary Figure 19: Long D4Z4 repeat alleles in GA4K can range from 10 kbp up to 100 kbp in length.

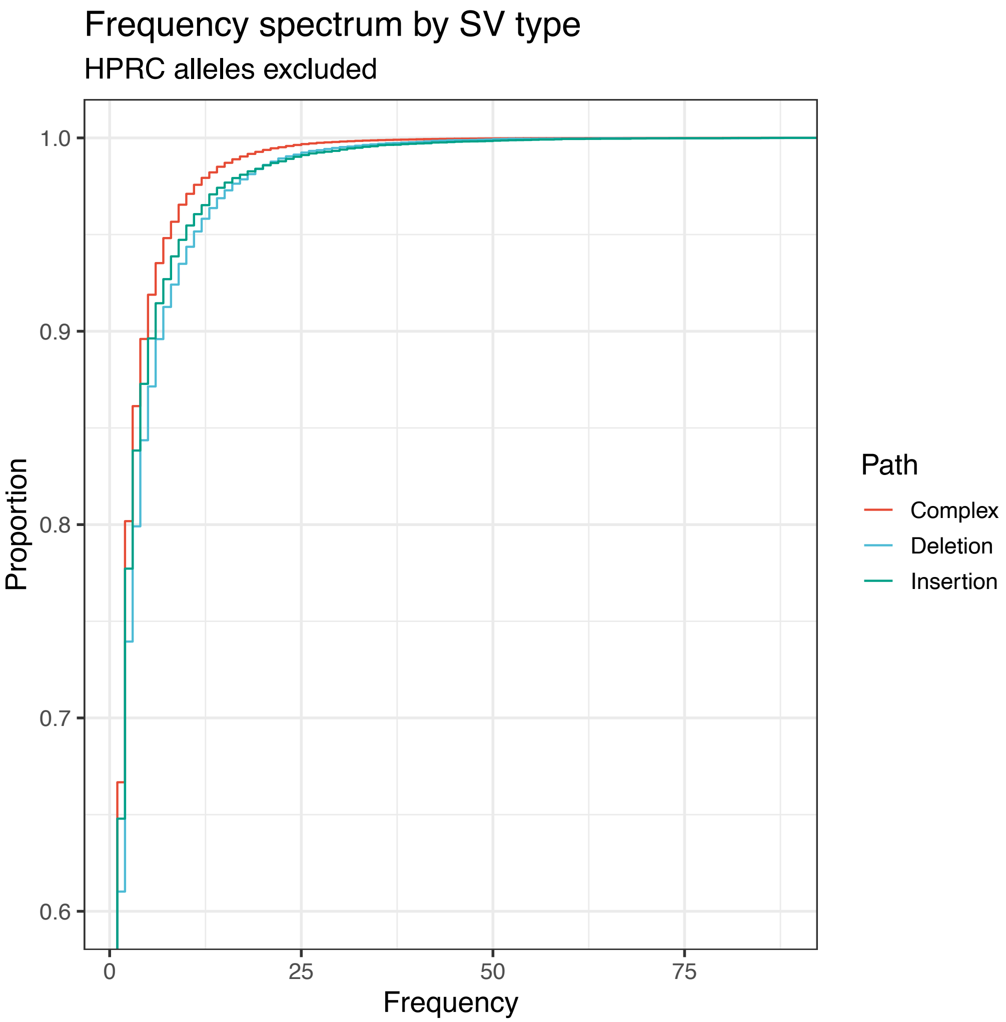

Supplementary Figure 20: SV frequency spectrum stratified by type.

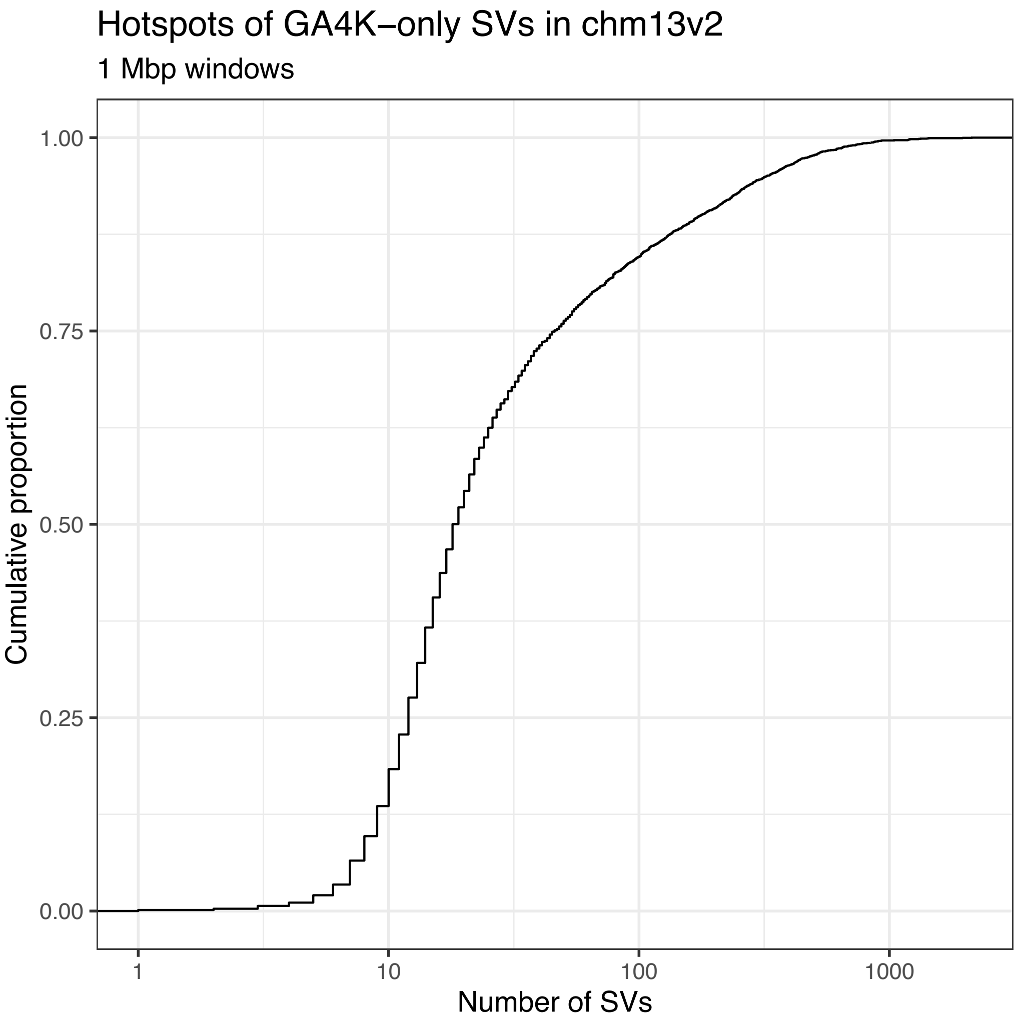

Supplementary Figure 21: Density of GA4K-only SVs in 1 Mbp windows across the genome.

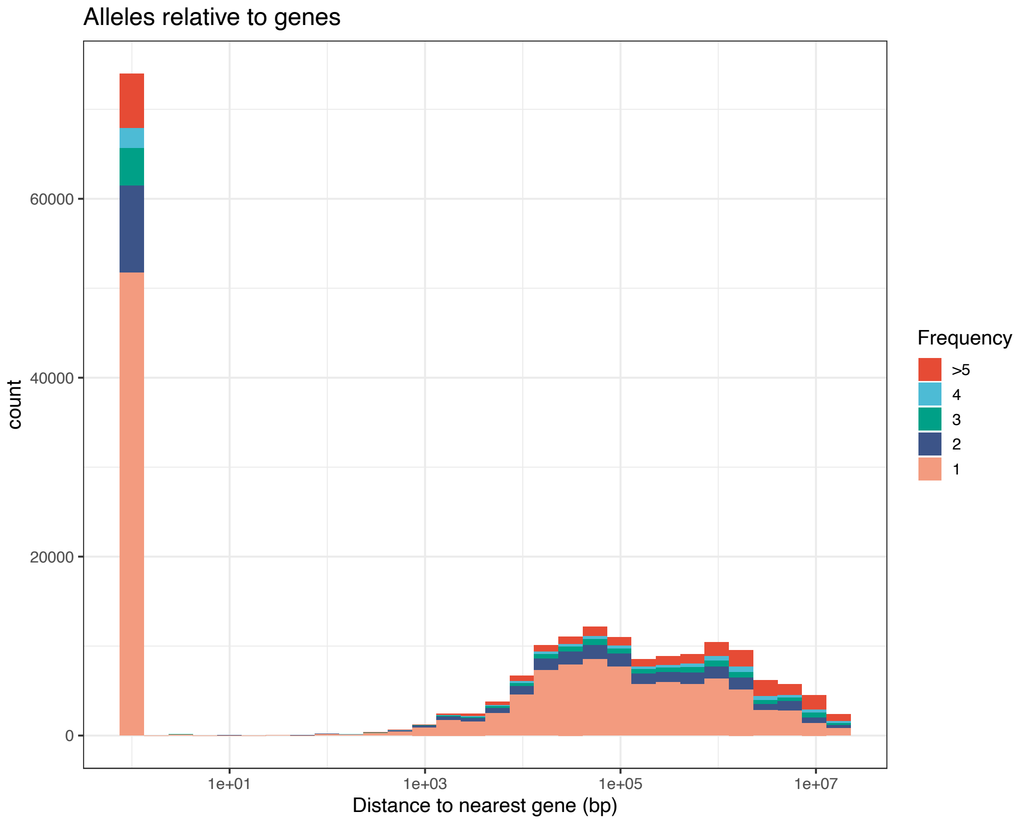

Supplementary Figure 22: Distance distribution of SV alleles that are unique to GA4K from the nearest gene, stratified by allele frequency.

Pathogenic variant in *AARS2*

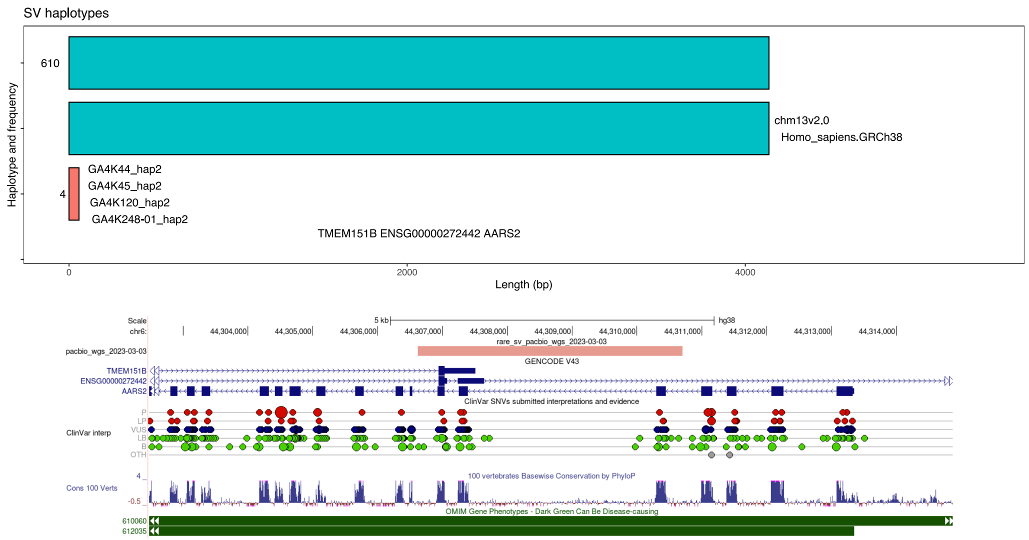

Supplementary Figure 23: Pathogenic variant in *AARS2*. Showing minigraph haplotype structures with frequencies and the UCSC genome browser view of the overlapping PBSV calls.

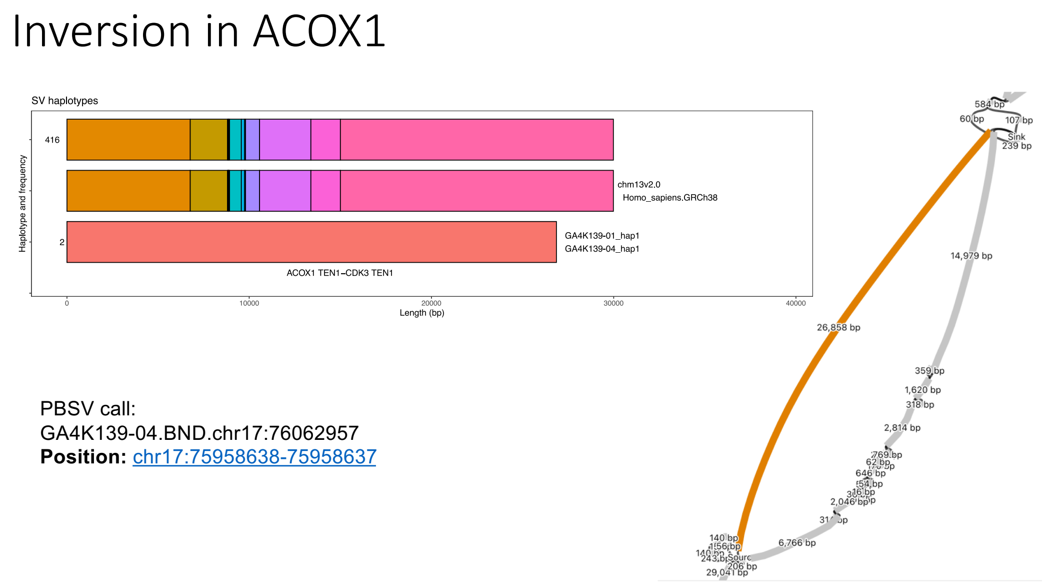

Supplementary Figure 24: Inversion in the *ACOX1* gene. Showing minigraph haplotype structures with frequencies. Each haplotype is an ordering of nodes. The minigraph bubble representing the inversion is also shown.

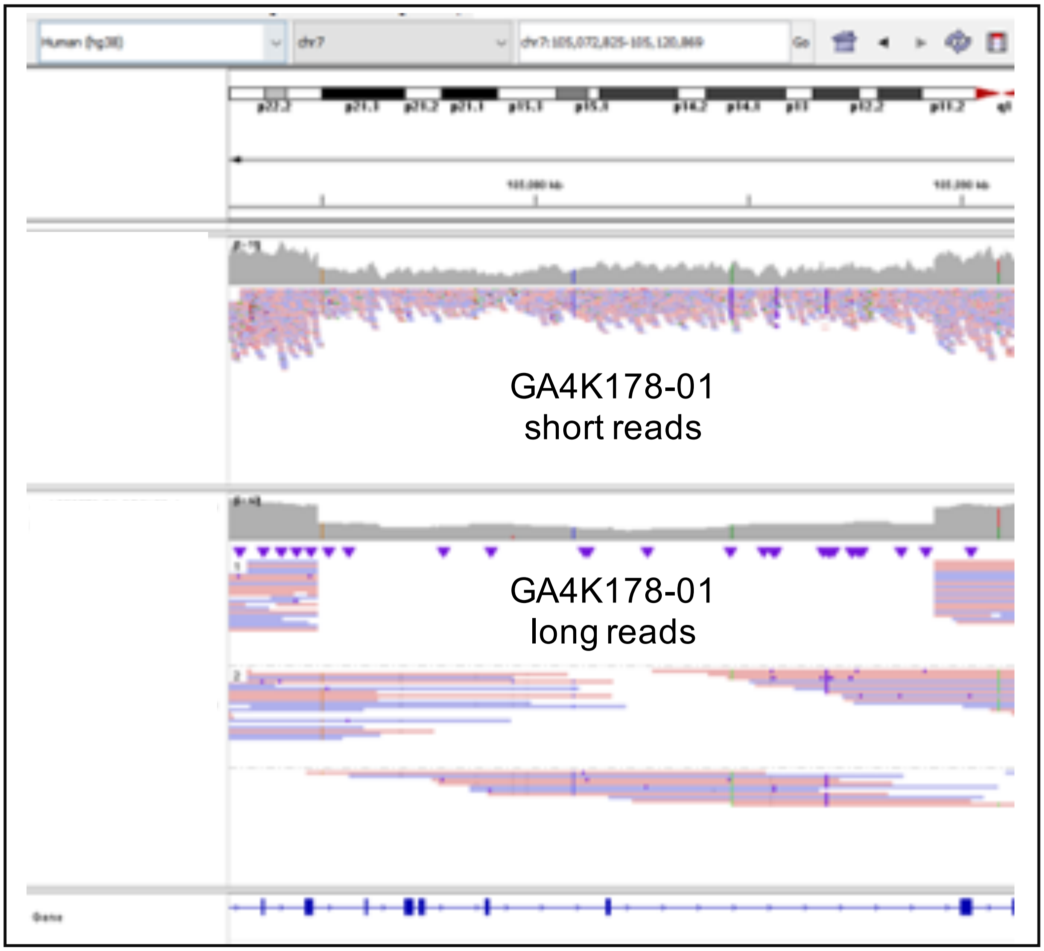

Supplementary Figure 25: Whole genome sequencing with short and long reads of the *KMT2E* deletion in GA4K178-01 viewed in the Integrated Genomics Viewer (IGV). Initially called by Dragen 3.6.3 but was not flagged since the OMIM gene field was missing from AnnotSV likely due to late addition of the gene to OMIM (original report of *KMT2E* related disease or O'Donnell-Luria-Rodan syndrome/OMIM:618512, was in 2019). This family was then routed to HiFi-GS where the diagnostic variant was detected and reported.

Supplementary Table 1: Number of contigs in GA4K86-01 diploid assembly that are not anchored in the genome graph but that are covered by short reads originating from the trio.

Mean bp	Maternal Assemblies	Paternal Assemblies
Total Assembly Size	3025967107.0	2899273161.5
Total Unanchored Size	17425226.0	26779214.5
Short-read covered both parents	16130019.0	23518471.5
Short-read maternal-only	952199.5	14706.5
Short-read paternal-only	43020.0	2572237.5

Supplementary Table 2: Mendelian violations of SVs called with PBSV and minigraph in the GA4K232 GA4K trio. Percentages are fractions of the total number of SVs called in the trio by minigraph or PBSV respectively.

Caller	Mendelian violations	Homozygous SVs in parents that should be in proband	SVs found in proband but absent in parents
PBSV	6002 (19.9%)	2293 (7.61%)	3709 (12.3%)
Minigraph	4978 (11.1%)	1601 (3.57%)	3377 (7.53%)

Supplementary Table 3: Precision of SV calls on HG001 benchmark with minigraph, PBSV, and their consensus.

Call set	Number of calls	Precision
Minigraph	24239	89.9%
PBSV	22613	84.9%
Minigraph PBSV consensus	20104	94.8%

Supplementary Table 4: Composition of SV concordant and discordant sets and the number of SVs recovered by Illumina Manta SV calls in the concordant/discordant sets (Methods).

Rare (5%) PacBio/Illumina SVs, rare (5%) 2-allele MG calls						
MG alleles	PB SVs	ILMN SVs	Concordant SVs	Discordant SVs	Concordant rep #	Discordant rep #
679	3064	4357	185	2879	112 (61%)	229 (8%)

All PacBio/Illumina SVs, 2-allele MG calls						
MG alleles	PB SVs	ILMN SVs	Concordant SVs	Discordant SVs	Concordant rep #	Discordant rep #
95594	22205	11610	5448	16757	3060 (56%)	5503 (33%)
