# Supplementary material for: Pangenome graphs improve the analysis of structural variants in rare genetic diseases
Source: Nat Commun. 2024 Jan 22;15:657. doi: 10.1038/s41467-024-44980-2 (PMC10803329; doi:10.1038/s41467-024-44980-2)
Supplement: Supplementary file 3 — Description of Additional Supplementary Files [file 41467_2024_44980_MOESM3_ESM.pdf]

## Description of Additional Supplementary Files

**File name: Supplementary Data 1**

**Description:** List of previous clinical assays that were performed for each proband, including exomesequencing (ES), whole genome sequencing (WGS) and chromosomal microarray (CMA).

**File name: Supplementary Data 2**

**Description:** Summary of leading rare GA4K alleles in phrank rankings and that map to OMIM genes, together with their pathogenic potential.

**File name: Supplementary Data 3**

**Description:** Sex and age reporting for study participants.
