# Supplementary material for: Pangenome graphs improve the analysis of structural variants in rare genetic diseases
Source: Nat Commun. 2024 Jan 22;15:657. doi: 10.1038/s41467-024-44980-2 (PMC10803329; doi:10.1038/s41467-024-44980-2)
Supplement: Supplementary file 7 — Reporting Summary [file 41467_2024_44980_MOESM7_ESM.pdf]

nature portfolio

Corresponding author(s): Tomi Pastinen, Guillaume Bourque

Last updated by author(s): 12/12/2023

Reporting Summary

Nature Portfolio wishes to improve the reproducibility of the work that we publish. This form provides structure for consistency and transparency in reporting. For further information on Nature Portfolio policies, see our Editorial Policies and the Editorial Policy Checklist.

Statistics

For all statistical analyses, confirm that the following items are present in the figure legend, table legend, main text, or Methods section.

n/a	Confirmed	
☐	☒	The exact sample size (*n*) for each experimental group/condition, given as a discrete number and unit of measurement
☐	☒	A statement on whether measurements were taken from distinct samples or whether the same sample was measured repeatedly
☐	☒	The statistical test(s) used AND whether they are one- or two-sided<br>*Only common tests should be described solely by name; describe more complex techniques in the Methods section.*
☐	☒	A description of all covariates tested
☐	☒	A description of any assumptions or corrections, such as tests of normality and adjustment for multiple comparisons
☐	☒	A full description of the statistical parameters including central tendency (e.g. means) or other basic estimates (e.g. regression coefficient) AND variation (e.g. standard deviation) or associated estimates of uncertainty (e.g. confidence intervals)
☒	☐	For null hypothesis testing, the test statistic (e.g. *F*, *t*, *r*) with confidence intervals, effect sizes, degrees of freedom and *P* value noted<br>*Give P values as exact values whenever suitable.*
☒	☐	For Bayesian analysis, information on the choice of priors and Markov chain Monte Carlo settings
☒	☐	For hierarchical and complex designs, identification of the appropriate level for tests and full reporting of outcomes
☒	☐	Estimates of effect sizes (e.g. Cohen's *d*, Pearson's *r*), indicating how they were calculated

Our web collection on statistics for biologists contains articles on many of the points above.

Software and code

Policy information about availability of computer code

Data collection	No software was used for data collection.
Data analysis	For trio graph binning, unique parental k-mers were counted with yak count v.01.<br>Proband HiFi reads were assembled with hifiasm v0.15 in trio mode to produce haplotype resolved assemblies.<br>Haplotype resolved assemblies were validated using the Secphase-Flagger pipeline (https://dockstore.org/workflows/github.com/mobinasri/flagger/Flagger:v0.1?tab=info)<br>The pangenome was constructed using mingraph -0.20 (r559) using the command minigraph -xggs -c with base-level alignments enabled on top of the chm13v2.0 backbone reference.<br>Each proband was genotyped on the pangenome using minigraph call -xasm --call.<br>The sequence nodes in the pangenome were annotated with RepeatMasker (version open-4.0.7) using the Dfam2 repeat database.<br>Nodes were aligned to the reference using minimap2 with the short reads preset.<br>We used phrank to rank SVs based on their overlap with gene annotations.<br>We used PBVS to call SVs from long-read alignments to GRCh38.<br>We used SURVIVOR to merge reference-based SV calls across multiple genomes.<br>We used Illumina Manta to call SVs from short read alignments to GRCh38.<br>We used PacBio lima and isoseq refine to analyze the Iso-Seq RNA.<br>Read depth was calculated with mosdepth.<br>For two trios, the short read sequencing data was aligned with DRAGEN.

For manuscripts utilizing custom algorithms or software that are central to the research but not yet described in published literature, software must be made available to editors and reviewers. We strongly encourage code deposition in a community repository (e.g. GitHub). See the Nature Portfolio guidelines for submitting code & software for further information.

Data

Policy information about availability of data

All manuscripts must include a data availability statement. This statement should provide the following information, where applicable:

- Accession codes, unique identifiers, or web links for publicly available datasets
- A description of any restrictions on data availability
- For clinical datasets or third party data, please ensure that the statement adheres to our policy

The 5-base HiFi-GS, HiFi long-read transcript sequencing (IsoSeq) and WGBS raw and processed data generated in this study have been deposited in the dbGAP (https://www.ncbi.nlm.nih.gov/gap/) database under accession code phs002206.v4.p1 [https://www.ncbi.nlm.nih.gov/projects/gap/cgi-bin/study.cgi?study_id=phs002206.v4.p1]. Raw and processed data are available under restricted access due to IRB regulations and informed consent limiting access to users studying genetic diseases. Data access is provided by dbGAP (https://dbgap.ncbi.nlm.nih.gov/aa/wga.cgi?page=login) for certified investigators with local IRB approval in place. The CHM13v2.0 reference genome is available for download at https://s3-us-west-2.amazonaws.com/human-pangenomics/T2T/CHM13/assemblies/analysis_set/chm13v2.0.fa.gz and the GRCh38 reference genome is available for download at https://hgdownload.soe.ucsc.edu/goldenPath/hg38/chromosomes/.
The GA4K genome graph, allele definitions and their frequencies, together with related data on assembly size, read depth, validation with Flagger and RepeatMasker results are available for download at https://doi.org/10.5281/zenodo.8309976.

Research involving human participants, their data, or biological material

Policy information about studies with human participants or human data. See also policy information about sex, gender (identity/presentation), and sexual orientation and race, ethnicity and racism.

Reporting on sex and gender	Sex is reported in Supplementary Data 3 for all study participants and assigned from self-reporting at enrollment and confirmed with genomic analysis. We focus all analysis on autosomes thus the findings are applicable to both sexes and no sex-specific analysis was performed.
Reporting on race, ethnicity, or other socially relevant groupings	Race and ethnicity were self-reported by the participants.<br>We do not use self reported race, ethnicity or other socially relevant groupings in our analyses. However, we did correlate the population structure of genotypes using Somalier on SNPs from Illumina GSA array and compared these results with the population structure obtained on structural variant genotypes as a validation of appropriate accounting of genetic ancestry from HiFi-GS assemblies.
Population characteristics	The study cohort described includes 1243 affected probands from 1078 families, with a total of 1367 individuals (detailed in Supplemental Data 1) enrolled in the Genomic Answers for Kids program. Probands age at enrollment ranged from 0 to 32 years (median 6 years) with 47% being female and 53% male, respectively.
Recruitment	A patient is considered eligible for the study if clinical genetic testing is indicated or was previously completed, or if they have a suspected genetic diagnosis based on clinical presentation and/or an existing molecular or cytogenetic finding. Providers introduce the study and ask if the family is interested in the study. If the family is interested a study team member contacts the patient to arrange for informed consent. If possible, the family will be consented during their clinic appointment. If consent during the clinic appointment is not possible, the patient is contacted and enrolled over the phone at a time that is convenient. Study coordinators access the medical record and extract clinical information which will be kept indefinitely. Families may also self-refer using a QR code provided on study marketing materials, or by e-mailing the study group directly. Self-referred patients are screened for eligibility by a genetic counselor (GS) or similarly qualified study member to ensure no self-selection bias exist. If a self-referred patient does not qualify for the study based on clinical assessment by the GC or qualified study member, the family will be notified and encouraged to recontact the study if the patient's clinical presentation changes in the future. Self-referred patients who qualify for the study will be contacted and enrolled in the same way as those patients referred by a provider. If the family decides to no longer be part of the study, their information will be de-identified, but their de-identified information will remain.
Ethics oversight	The study complies with all relevant ethical regulations as approved by the Children's Mercy Institutional Review Board (IRB) (Study # 11120514). Informed written consent was obtained from all participants prior to study inclusion and included consenting for collecting biospecimens for the purpose of deriving patient-specific cell lines. Participants were not compensated for study participation.

Note that full information on the approval of the study protocol must also be provided in the manuscript.

Field-specific reporting

Please select the one below that is the best fit for your research. If you are not sure, read the appropriate sections before making your selection.

☒ Life sciences ☐ Behavioural & social sciences ☐ Ecological, evolutionary & environmental sciences

For a reference copy of the document with all sections, see nature.com/documents/nr-reporting-summary-flat.pdf

Life sciences study design

All studies must disclose on these points even when the disclosure is negative.

Sample size	No statistical method were used to predetermine the sample size. Our study does not perform statistical tests on these genomes or try to find statistical associations between different variables involving these genomes. Also, we do not attempt to generalize to a larger population of genomes from this sample.
Data exclusions	No sample or data sets were excluded
Replication	We validated the genome assemblies with Flagger and excluded structural variant calls that are not supported by a reliable assembled sequence. The genomes in this cohort were sequenced and assembled only once.
Randomization	No experimental groups were included, thus no randomizations were done
Blinding	Family-based (trio) analysis is key for the identification of disease variants in suspected rare disease, thus no blinded analysis was performed. It is otherwise difficult or entirely impossible to identify rare disease variants if blinding is applied. Therefore, blinding does not apply in this context.

Reporting for specific materials, systems and methods

We require information from authors about some types of materials, experimental systems and methods used in many studies. Here, indicate whether each material, system or method listed is relevant to your study. If you are not sure if a list item applies to your research, read the appropriate section before selecting a response.

Materials & experimental systems

n/a	Involved in the study
☒	☐ Antibodies
☒	☐ Eukaryotic cell lines
☒	☐ Palaeontology and archaeology
☒	☐ Animals and other organisms
☒	☐ Clinical data
☒	☐ Dual use research of concern
☒	☐ Plants

Methods

n/a	Involved in the study
☒	☐ ChIP-seq
☒	☐ Flow cytometry
☒	☐ MRI-based neuroimaging
